# Short-Term Fertilization with the Nitrogen-Fixing Bacterium (NFB) *Kosakonia radicincitans* GXGL-4A Agent Can Modify the Transcriptome Expression Profiling of Cucumber (*Cucumis sativus* L.) Root

**DOI:** 10.3390/microorganisms13030506

**Published:** 2025-02-25

**Authors:** Baoyun Feng, Erxing Wang, Yating Zhang, Lurong Xu, Yanwen Xue, Yunpeng Chen

**Affiliations:** 1 Department of Resources and Environment, School of Agriculture and Biology, Shanghai Jiao Tong University, Shanghai 200240, China; 17750205452@163.com (B.F.); wex.666@sjtu.edu.cn (E.W.); zyting2022@sjtu.edu.cn (Y.Z.); 2 Asset Management and Shared Equipment’s Office, School of Agriculture and Biology, Shanghai Jiao Tong University, Shanghai 200240, China; lurongxu@sjtu.edu.cn (L.X.); xueyanwen@sjtu.edu.cn (Y.X.); 3 Shanghai Yangtze River Delta Eco-Environmental Change and Management Observation and Research Station (Shanghai Urban Ecosystem Research Station), Ministry of Science and Technology, National Forestry and Grassland Administration, 800 Dongchuan Rd., Shanghai 200240, China

**Keywords:** qRT-PCR, nitrogen-fixing bacterium, nitrate transport gene, electrical conductivity, differential transcriptome, *Cucumis sativus*

## Abstract

The application of nitrogen-fixing bacteria (NFB) as a biofertilizer can greatly reduce or even avoid environmental pollution caused by the excessive use of chemical nitrogen fertilizers. To explore the effect of short-term fertilization of GXGL-4A on the expression of functional genes in the roots of the cucumber (*Cucumis sativus* L.) cultivar “Xintaimici”, this study used transcriptome sequencing technology combined with fluorescent quantitative RT-PCR (qRT-PCR) verification to compare the gene transcription profiles of GXGL-4A-treated and control (sterile-water-treated) groups. A total of 418 differentially expressed genes (DEGs) were detected. The transcription levels of genes *Csa5G161290* and *Csa3G027720*, which encode nitrate transporters, showed significant up-regulation (3.04- and 2.27-fold, respectively) in roots inoculated with GXGL-4A. The genes *CsaV3_5G006200*, encoding cytokinin dehydrogenase involved in the biosynthesis of zeatin, *CsaV3_1G011730*, encoding a wound-responsive protein, and *CsaV3_6G015610*, encoding a heat stress transcription factor, were significantly up-regulated at the transcriptional level (*p* < 0.05). However, the transcription of nitrogen cycling functional genes *CsaV3_3G036500*, *CsaV3_1g008910*, and *CsaV3_3G018610*, which encode nitrate reductase, high-affinity nitrate transporter (NRT), and ferredoxin-nitrite reductase, respectively, showed significant down-regulation (*p* < 0.05). Only the KEGG pathway of phenylpropanoid biosynthesis reached a significant level (*p* < 0.05). This study contributes to a deeper understanding of the interaction between NFB and plants and provides theoretical guidance for the development of GXGL-4A as a mature biological agent for sustainable agricultural production under drought stress.

## 1. Introduction

Cucumber (*Cucumis sativus* L.) is a worldwide cultivated vegetable and, since 1970, due to the establishment of protected field cultivation facilities, the cultivated area of cucumber has been increasing continuously. According to FAO statistics, as of 2019, the world’s cucumber cultivation area and total yield reached 2,231,402 hectares and 87,805,086 tons, respectively. Whether it is protected cultivation or open-field cultivation, there is excessive use of chemical nitrogen fertilizers in cucumber production, which will lead to the accumulation of nitrite in cucumber, thus affecting its commodity quality. Moreover, the unreasonable use of nitrogen fertilizers in farmland ecosystems may result in the eutrophication of water, soil acidification, and other environmental problems [1,2,3]. Thus, improving the nitrogen use efficiency of crops and reducing the environmental pollution caused by chemical nitrogen fertilizers has become an urgent problem.

Plant roots can absorb various forms of nitrogen, including nitrate nitrogen, ammonium nitrogen, and some organic nitrogen compounds. Among them, the most important source of nitrogen in well-ventilated soil is nitrate nitrogen. Nitrate nitrogen is an essential nutrient for plant growth and development, and it also participates in signal regulation in plants [4]. The absorption, transportation, and assimilation of nitrate by higher plants mainly rely on nitrate transporters. In addition, nitrate reductase also plays a key role in nitrate assimilation [5]. When the external nitrate concentration is high, the low-affinity nitrate transport system (LATS, NRT 1 family) plays a major role. However, when the concentration of external nitrate is low, the high-affinity nitrate transporters (HATS, NRT 2 family) become active [6]. Nitrate transporters can be divided into constitutive and inducible types based on whether their biosynthesis is induced by external nitrate concentration. Scientists have reported that many constitutive high-affinity nitrate transporters can be highly expressed, even in the absence of nitrate [7]. To date, a total of 60 *nrt* genes have been found in *Arabidopsis*, of which 53 belong to the NRT 1 family and 7 belong to the NRT 2 family. Among the *Arabidopsis thaliana nrt* genes with proven functions, genes encoding proteins At NRT 1.1, At NRT 2.1, At NRT 2.2, and At NRT 2.5 are inducible, and the other *nrt* genes encoding proteins At NRT 1.2, At NRT 1.4, At NRT 2.3, At NRT 2.6, and At NRT 2.7 are constitutive [8]. At NRT 1.1 belongs to the dual-affinity nitrate transport system. The expression of At NRT 2.5 is inhibited by nitrate. Compared with research on the model plant *Arabidopsis thaliana*, the current research on the molecular regulation mechanism of low nitrogen-tolerance-related genes in cucumber is still in its infancy.

The completion of cucumber genome sequencing work provides a good platform for the construction of large-scale cucumber genetic maps and gene cloning. Many studies have shown that adversity stresses, including biological and abiotic factors, can affect the absorption, transportation, and assimilation of nitrogen in cucumber. For example, under low-temperature conditions, even if the nitrogen supply is sufficient, the absorption of nitrate nitrogen by cucumber roots decreases significantly. Cucumber is a typical nitrate-preferred plant. When ammoniacal nitrogen is used as the only nitrogen source, the growth of cucumber seedlings is inhibited, and the plant will even wither in severe cases [9,10]. It has been reported that the application of nitrogen fertilizer can significantly increase the absorption of calcium, magnesium, and zinc by cucumber, thus greatly enhancing the yield of cucumber [11]. In the past few years, the low-affinity nitrate transporter gene of cucumber was introduced into an *Arabidopsis* mutant, and its nitrate absorption ability was successfully restored [12]. Zhao et al. analyzed the transcriptome data of cucumber under nitrogen-deficient conditions and found that the transcription factor MYB12 can regulate auxin and ethylene signals, thereby promoting the absorption of nitrate by cucumber under nitrogen-deficient conditions [13]. Although the physiological and biochemical responses of cucumber to different nitrogen nutrition levels have been extensively investigated, most studies still focus on the effects of nitrogen forms and levels on plant growth [14,15]. The promoting effect of NFB on plant growth is well known; however, the impact of NFB application on plant root transcriptomes is still unclear.

Extensive use of nitrogen (N) fertilizer boosts yields but risks environmental issues such as eutrophication via N leaching into groundwater and agricultural runoff into surface water and soil acidification [16,17]. When addressing these environmental problems, the establishment of an effective governance mechanism is particularly important. The application of NFB has been considered as a promising strategy to deal with these issues [18,19]. At present, research on biological nitrogen fixation (BNF) mainly includes the interactions between legumes and rhizobia, isolation of NFB strains, synthetic biological modification of nitrogen-fixing microorganisms and the interaction between NFB and microalgae [20,21]. The effect of NFB agent on nitrate absorption and transport in cucumber roots has not been studied. To examine the responses of cucumber plants to a short-term NFB application and deeply understand the interactions between NFB bacterial cells and cucumber roots, comparative transcriptome analysis of cucumber (*Cucumis sativus* L.) roots in response to NFB treatment was conducted. The expression differences of some important functional genes related to nitrate transport, hormone synthesis, innate immunity, and abiotic stress resistance in plants at a transcription level were verified by fluorescent quantitative RT-PCR (qRT-PCR).

## 2. Materials and Methods

### 2.1. Cucumber Cultivar and NFB Strain

The seeds of cucumber (*Cucumis sativus* L.) variety “Xintaimici” were purchased from the Original Seed Farm in Xintai City, Shandong Province of China. The associative nitrogen-fixing bacterium (NFB) GXGL-4A isolated from maize (*Zea mays* L.) roots and its Tn5 mutants with different siderophore-producing capabilities M107 and M246-2 were used for a short-term inoculation on cucumber seedlings in this study [22,23].

### 2.2. Cucumber Culture System

The cultivation of cucumber was carried out according to the following procedures. Cucumber seeds were surface-sterilized with 75% ethanol for 30 s and then soaked in 1% sodium hypochlorite for about 10 min and, finally, washed thoroughly with sterile water. The treated seeds were subjected to germination in an incubator at 37 °C. Subsequently, the germinated cucumber seeds were transferred to a closed sterile beaker (10.5 cm in diameter and 14.5 cm in height) and sown on a large piece of sterile degreasing cotton ball to fix the cucumber seedlings. The germinated seeds were divided into two groups. One group was hydroponically cultivated with sterile nitrate-free Hoagland nutrient solution (CK group), and the other group was cultured in nitrate-free Hoagland solution supplied with NFB bacterial cells of GXGL-4A (T group). The Hoagland nutrient solution was prepared with reference to the literature [24]. Considering that the NFB bacterial cells of GXGL-4A can provide a nitrogen source for plant growth via BNF and, more importantly, to eliminate the impact of nitrate nitrogen in original Hoagland nutrient solution on plant growth and cucumber root transcriptome, nitrate was not added in the nutrient solution in this study. Except for this point, other composition concentrations were consistent with the literature description. The cucumber seedlings that were fixed with degreasing cotton balls grew in the modified Hoagland nutrition solution.

### 2.3. Treatments of Cucumber Seedlings

Bacterial cells of GXGL-4A were inoculated in the rhizosphere of cucumber seedlings when cotyledons were fully expanded. The bacterial cells of GXGL-4A were inoculated into LB medium (tryptone 10 g, yeast extract 5 g, and sodium chloride 10 g) at a volume ratio of 1% and cultured overnight at 37 °C in a shaker. Then, cells were collected after a centrifugation at 8000 rpm for 8 min and resuspended with sterile water. After two rounds of centrifugation and rinsing, the bacterial precipitate was resuspended with the same volume of sterile water. A total of 10 mL of GXGL-4A cell suspension (about 1 × 10^8^ cfu/mL) was released into the rhizosphere of each cucumber seedling cultivated in 10 mL of Hoagland nutrient solution (nitrate-free) (T group). In the CK group, cucumber seedlings were treated with 10 mL of sterile water. All the cucumber seedlings were treated once every 3 days and carefully taken out after three treatments. A total of around 0.3 g of cucumber roots in each group was promptly harvested and placed in 1.5 mL Eppendorf tubes (about 0.1 g per tube). Subsequently, the roots were stored in liquid nitrogen for later RNA isolation.

### 2.4. Library Preparation and Illumina HiSeq XTen/NovaSeq 6000 Sequencing

Total RNA was extracted from the cucumber roots using TRIzol^®^ reagent (Plant RNA Purification Reagent for plant tissue) according to the manufacturer’s instructions (Invitrogen, Carlsbad, CA, USA), and genomic DNA was removed using DNase I (TaKara, Osaka, Japan). Then, RNA quality was determined by 2100 Bioanalyzer (Agilent, Santa Clara, CA, USA) and quantified using the ND-2000 (NanoDrop Technologies, Wilmington, DE, USA). Only a high-quality RNA sample (OD260/280 = 1.8~2.2, OD260/230 ≥ 2.0, RIN ≥ 6.5, 28S:18S ≥ 1.0, >1 μg) was used to construct the sequencing library.

RNA-seq transcriptome libraries of the T and CK groups were established according to the manual of TruSeqTM RNA sample preparation Kit from Illumina (San Diego, CA, USA) using 1 μg of total RNA. Shortly, the messenger RNA was isolated according to the polyA selection method by oligo (dT) beads and then fragmented by fragmentation buffer firstly. Secondly, the double-stranded cDNA was synthesized using a Superscript double-stranded cDNA synthesis kit (Invitrogen, CA) with random hexamer primers (Illumina). Then, the synthesized cDNA was subjected to end-repair, phosphorylation, and “A” base addition according to Illumina’s library construction protocol. Libraries were size selected for cDNA target fragments of 300 bp on 2% Low-Range Ultra Agarose followed by PCR amplification using Phusion DNA polymerase (NEB) for 15 PCR cycles. After a quantification by TBS380, the paired-end RNA-seq sequencing library was sequenced on the Illumina HiSeq XTen/NovaSeq 6000 platforms (2 × 150 bp read length).

### 2.5. Read Mapping

The raw paired-end reads were trimmed and quality-controlled by SeqPrep (https://github.com/jstjohn/SeqPrep accessed on 25 August 2020) and Sickle (https://github.com/najoshi/sickle accessed on 25 August 2020) with default parameters. Then, the clean reads were separately aligned to the reference genome of cucumber “Chinese long” with orientation mode using HISAT2 (version 2.1.0, http://ccb.jhu.edu/software/hisat2/index.shtml accessed on 25 August 2020) software [25]. The mapped reads of each sample were assembled by StringTie (version 2.1.2, https://ccb.jhu.edu/software/stringtie/index.shtml?t=example accessed on 25 August 2020) in a reference-based approach [26].

### 2.6. Differential Expression Analysis and Functional Enrichment

To identify the differentially expressed genes (DEGs) between samples, the expression level of each transcript was calculated according to the transcripts per million reads (TPM) method. RSEM (version 1.3.3, http://deweylab.biostat.wisc.edu/rsem/ accessed on 25 August 2020) was used to quantify gene abundances [27]. Differential expression analysis was performed using software, i.e., DESeq2 and the EdgeR with Q value ≤ 0.05, and the DEGs with |log2FC| > 1 and Q value ≤ 0.05 were significantly different at a transcriptional level [28,29]. In addition, the functional-enrichment analysis, including GO and KEGG, was conducted to identify those DEGs significantly enriched in GO terms and metabolic pathways at a Bonferroni-corrected *p*-value (≤0.05) compared with the whole-transcriptome background. Eventually, GO functional enrichment and KEGG pathway analyses were carried out by Goatools (version 0.6.5, https://github.com/tanghaibao/Goatools accessed on 25 August 2020) and KOBAS (version 2.1.1) [30].

### 2.7. The qRT-PCR Analysis of Functional Genes in Cucumber Root

The transcription levels of some representative functional genes in the roots of a cucumber cultivar “Xintaimici” were verified using fluorescent quantitative RT-PCR (qRT-PCR) after transcriptome analysis. PCR primer pairs were designed according to the genome sequence of a cucumber cultivar “Chinese Long” (ftp://cucubitgenomics.org/pub/cucurbit/genome/cucumber/Chinese_long/ accessed on 25 August 2020) [31] (Table 1).

Total RNA was isolated using the EZ-10 Spin Column Total RNA Isolation Kit (Sangon, Shanghai, China, the order No.: B610583). The first-strand cDNA synthesis was performed using Sangon biotech’s First Strand cDNA Synthesis Kit (Order NO.: B300537). All the experimental procedures were carried out according to the manufacturers’ instructions.

The transcription levels of functional genes selected in cucumber roots were determined with a housekeeping gene *β*-actin as internal control by qRT-PCR. The qRT-PCR reaction mixture consisted of CybrGreen qPCR Master Mix (High Rox, B639273, BBI) of 10 μL, forward and reverse primers of 0.4 μL each, 2 μL of cDNA template, and ddH_2_O of 7.2 μL. Real-time PCR was assayed in an ABI StepOnePlus thermocycler (ABI, Foster, CA, USA).

### 2.8. Statistical Analysis

The relative gene expression levels were calculated by the 2^−ΔΔCt^ square method, and statistical analysis of the data was conducted using the software IBM SPSS (version 17.0).

## 3. Results

### 3.1. Illumina RNA Sequencing (RNA-Seq) and Sequence Assembly

An RNA-Seq-based transcriptome profiling of nitrogen metabolism response under NFB treatment condition in cucumber roots was performed, and a total of 38.46 GB of clean data were obtained. The clean data of each sequencing sample were more than 6.07 GB, and the percentage of Q30 base was over 95.18%. Clean reads from each sequencing sample were blasted with the reference genome of *Cucumis sativus* 9930 (genome version: V3, http://cucurbitgenomics.org/organism/20 accessed on 25 August 2020), and the alignment rate ranged from 86.9% to 96.8%. The sequence data are deposited in NCBI Sequence Read Archive (SRA, http://www.ncbi.nlm.nih.gov/Traces/sra accessed on 28 September 2021) with the BioProject ID PRJNA766832. Overall, 21,158 unigenes (including 1037 unknown genes) and 38,569 expressed transcripts, including 19,275 known transcripts and 19,294 unknown transcripts, were detected.

The unigenes and transcripts were annotated by searching the reference sequences using BLASTX (version 2.9.0) against the major functional databases, i.e., NR (https://www.ncbi.nlm.nih.gov/refseq/about/nonredundantproteins/ accessed on 25 August 2020), Swiss-Prot (https://www.expasy.org/resources/uniprotkb-swiss-prot accessed on 25 August 2020), COG (https://www.ncbi.nlm.nih.gov/research/cog accessed on 25 August 2020), GO (https://geneontology.org/ accessed on 25 August 2020), Pfam (version 32), and KEGG (version 2018), and the statistics of annotation results for cucumber “Xintaimici” unigenes are shown in Figure S1 and Table S1. The assembly produced a substantial number of long transcripts: 1084 transcripts were <200 bp in length and 33,943 transcripts were >1000 bp. About half of the transcripts were between 200 and 1800 bp in length. A total of 21,552 transcripts were >1800 bp in length (Figure S2).

### 3.2. Expression Level Analysis of C. sativus Unigenes and Transcripts

The expression levels of unigenes and transcripts in cucumber (*C*. *sativus* L.) roots were analyzed quantitatively using the expression quantitative software RSEM (version 1.3.3). The quantitative index is TPM, which refers to taking the number of transcripts as the calculation unit and using the number of transcripts instead of the number of spliced fragments. Considering the length of transcripts and the number of unigenes expressed in samples, the quantification is more accurate under certain conditions, especially when the total number of genes expressed among samples varies greatly. Eventually, a total of 24,317 unigenes/transcripts were obtained by BLAST against the genome of *C*. *sativus* 9930 (version no: V3) (http://cucurbitgenomics.org/organism/20 accessed on 25 August 2020). The expression distribution of unigenes or transcripts in each sample of the T group and the CK group was similar, and there was no significant difference (Figure S3). The common and unique expressed genes/transcripts among groups were analyzed. The result of Venn analysis showed that 15,832 genes/transcripts were detected in each group, and 463 specifically expressed genes/transcripts were found in the CK group and 540 unique expression genes were revealed in the T group.

### 3.3. Variation in Gene Expression Among Groups

**Figure 1:**
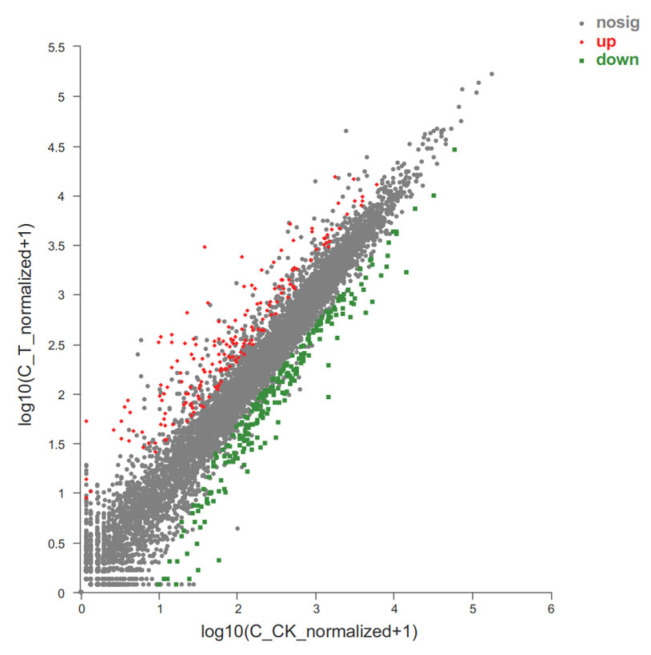
Differential expression of unigenes between groups. The abscissa and ordinate, respectively, represent the expression amount of unigene/transcript in the two samples. Here, the values of abscissa and ordinate are logarithmicized, and each point represents a specific unigene/transcript. The abscissa value corresponding to a specific point indicates the expression of the unigene in the control sample, and the ordinate value represents the expression of the gene in the treatment sample. The red dot in the figure indicates the significantly up-regulated unigenes, the green dot shows the significantly down-regulated unigenes, and the gray dot displays the nonsignificantly different unigenes. After mapping all unigenes, the closer the point to 0, the lower the expression. The greater the degree of deviation from the diagonal, the greater the difference in the expression of the unigene between two samples. The abbreviation “nosig” means no significance.

After the acquisitions of read/unigene/transcript counts, the differentially expressed genes (DEGs)/transcripts (DETs) between samples were identified using the software, DESeq2 and EdgeR (*p*-adjust < 0.05, |log2FC| ≥ 1). A total of 418 genes, including 174 up-regulated and 244 down-regulated genes, were detected in the T group in comparison with the CK group (Figure 1). The detailed gene expression differences are shown in Table S2.

### 3.4. GO Functional Classification of the DEGs

**Figure 2:**
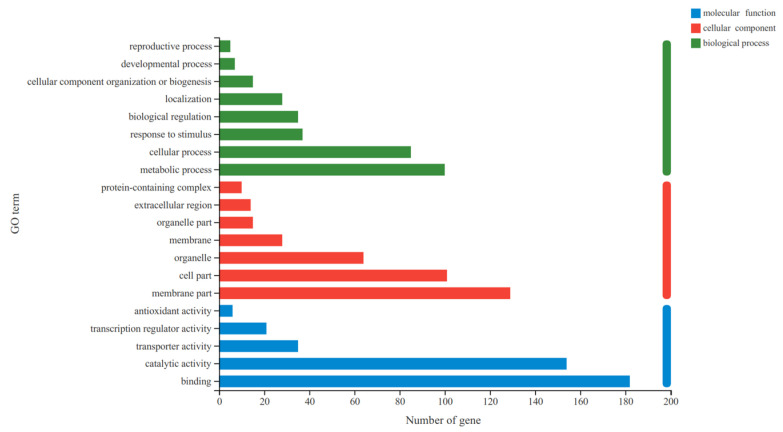
Gene ontology (GO) classification of the identified DEGs among groups. The ordinate in the figure represents the secondary classification terms of GO and the abscissa indicates the number of unigenes/transcripts in the secondary classification. Figure legend shows three major classifications.

Functional classification analysis was conducted based on the DEGs of *C*. *sativus*. In this study, 418 DEGs can be categorized into 20 functional groups. In the categories of biological process (BP), cellular component (CC), and molecular function (MF) of the GO classification, “metabolic process”, “membrane part”, and ”binding” were dominant. In addition, a high percentage of genes from the categories of “cellular process”, “cell part”, and “catalytic activity”, and a few genes from terms of “reproductive process”, “protein-containing complex”, and “antioxidant activity” were found (Figure 2).

### 3.5. Functional Classification of C. sativus DEGs by COG

To further explore the biological functions of DEGs of *C*. *sativus* between the T group and the CK group, the DEGs identified were categorized based on Clusters of Orthologous Groups of proteins (COG). All the DEGs are aligned to the COG database to predict and classify possible functions. Finally, 316 DEGs were assigned to 19 categories, and the cluster of “poorly characterized” (195, 61.71%) was the largest group, followed by “information storage and processing” (23, 7.28%), “cellular processes and signaling” (20, 6.33%), and “metabolism” (14, 4.43%) functional clusters (Table 2).

### 3.6. Functional Classification of C. sativus DEGs by KEGG Pathway Analysis

**Figure 3:**
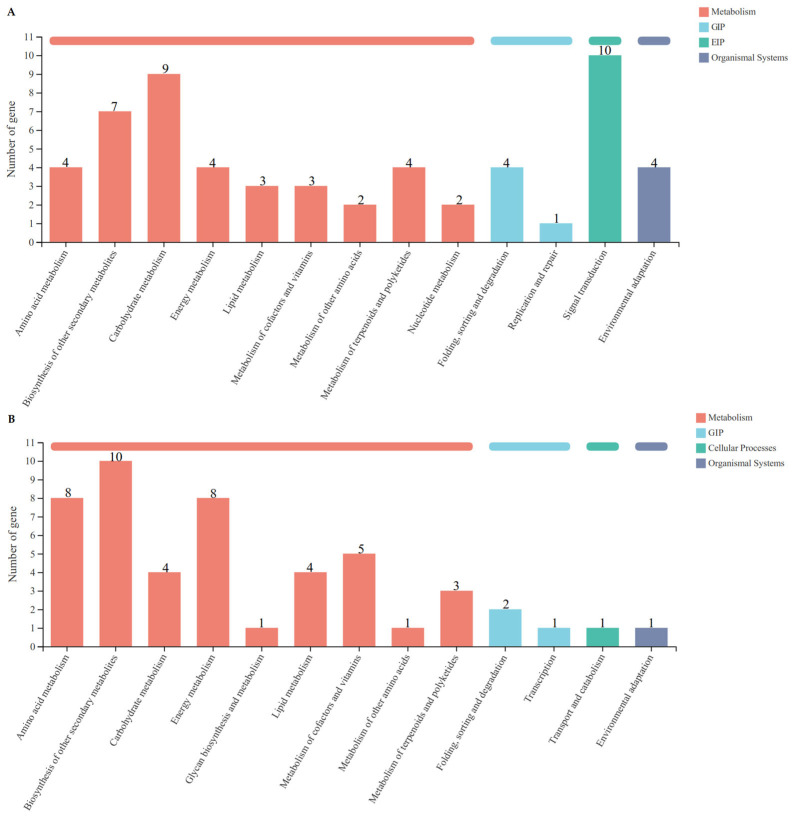
Functional classification of *C*. *sativus* DEGs by KEGG pathway analysis. The ordinate represents the name of KEGG metabolic pathway, and the abscissa indicates the number of up-regulated unigenes annotated to this pathway. GIP: genetic information processing; EIP: environmental information processing. (**A**): Up-regulated DEGs; (**B**): down-regulated DEGs.

To understand the biological pathways that might be active in *C*. *sativus*, the DEGs were compared against the Kyoto Encyclopedia of Genes and Genomes (KEGG) database. The results revealed that 57 of the 174 up-regulated genes were assigned to 13 KEGG pathways. The dominant KEGG pathways were signal transduction (10, 17.54%), carbohydrate metabolism (9, 15.79%), and biosynthesis of other secondary metabolites (7, 12.28%) (Figure 3A). Furthermore, 49 of 244 down-regulated genes were assigned to 13 KEGG pathways. The significantly dominant pathway was biosynthesis of other secondary metabolites (10, 20.41%), followed by the pathways of energy metabolism (8, 16.33%) and amino acid metabolism (8, 16.33%) (Figure 3B). The classification of KEGG pathways provides a valuable guide for exploring the important biological functions and metabolism pathways in *C*. *sativus* roots in response to the inoculation of NFB bacterial cells.

### 3.7. GO Enrichment Analysis of C. sativus DEGs

**Figure 4:**
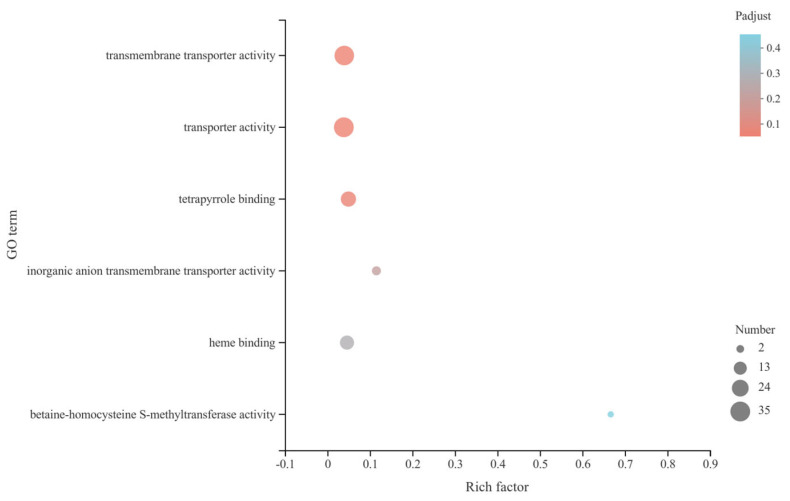
GO enrichments of DEGs in the roots of *C*. *sativus*. The vertical and horizontal axes, respectively, represent GO term and rich factor (the ratio of the number of genes enriched in the GO term to the number of annotated genes). The larger the rich factor, the greater the degree of enrichment. The size of every point means the number of genes in GO term, and the color of the point corresponds to *p*-adjust ranges.

GO enrichment analysis of *C*. *sativus* DEGs was carried out using the software Goatools to obtain the main GO functions of genes in the gene set [32]. Fisher tests were performed with the Goatools Python module. When the corrected *p* value (*p*-adjust) is less than 0.05, this GO function is considered significantly enriched. The results showed that DEGs were enriched in the categories of “transmembrane protein activity” (34, 29.82%), “transporter activity” (35, 30.70%), “tetrapyrrole binding” (20, 17.54%), “inorganic anion transmembrane transporter activity” (6, 5.26%), “heme binding” (17, 14.91%), and “betaine-homocysteine S-methyltransferase activity” (2, 1.75%). However, the functional enrichment levels of the GO terms were not significant (Figure 4).

### 3.8. KEGG Enrichment Analysis of C. sativus DEGs

**Figure 5:**
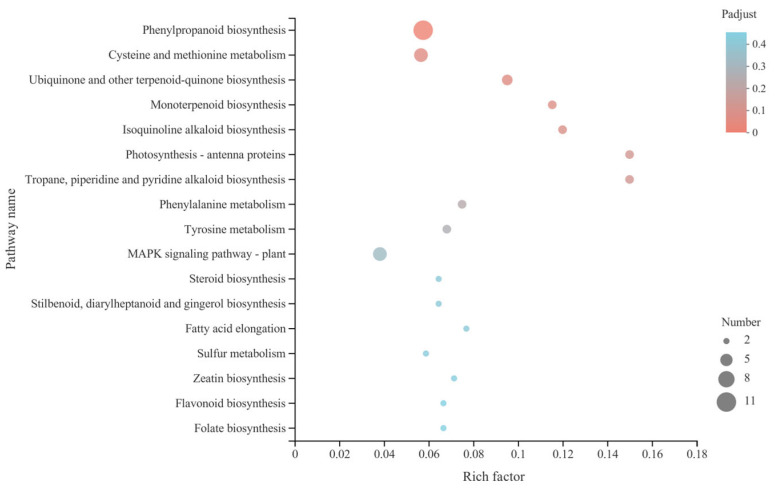
KEGG enrichments of the DEGs detected in the roots of *C*. *sativus*. The vertical and horizontal axes, respectively, represent KEGG pathway and rich factor (the ratio of the number of genes enriched in the KEGG pathway to the number of annotated genes). The larger the rich factor, the greater the degree of enrichment. The size of every point means the number of genes in the term of KEGG pathway, and the color of the point corresponds to *p*-adjust ranges.

The R script was used for the KEGG pathway enrichment analysis of *C*. *sativus* DEGs in the present study. When the *p*-adjust value is less than 0.05, the KEGG pathway is considered significantly enriched. The calculation principle is the same as that of GO functional enrichment analysis. Eventually, 59 DEGs were enriched into 17 KEGG pathways, including “phenylpropanoid biosynthesis” (11, 18.64%), “cysteine and methionine metabolism” (6, 10.17%), “MAPK signaling pathway-plant” (6, 10.17%), and “ubiquinone and other terpenoid-quinone biosynthesis” (4, 6.78%). It should be noted that only the KEGG pathway of phenylpropanoid biosynthesis was significantly enriched (*p*-adjust = 0.0279) (Figure 5).

### 3.9. Transcription Levels of the Genes Involeved in Nitrogen Metabolism in Cucumber Root

**Figure 6:**
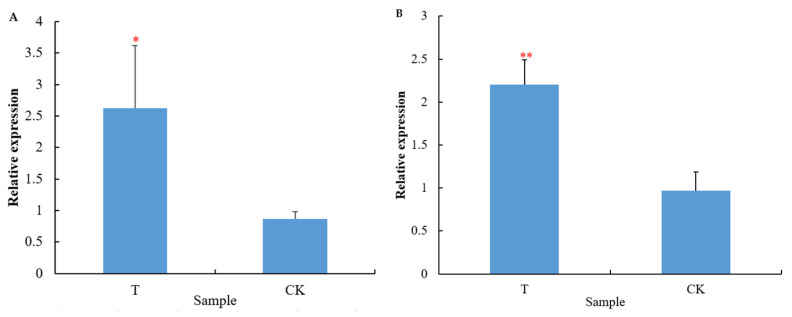
Transcription levels of NRT genes (**A**: *Csa5G161290*; **B**: *Csa3G027720*) in the cucumber roots between groups. A single red asterisk (*) indicates a significant difference at the transcription level (*p* < 0.05). Double red asterisks (**) represent an extremely significant difference at the transcription level (*p* < 0.01). T: cucumber roots treated with the bacterial cells of GXGL-4A; CK: cucumber roots treated with sterile water (the control).

**Figure 7:**
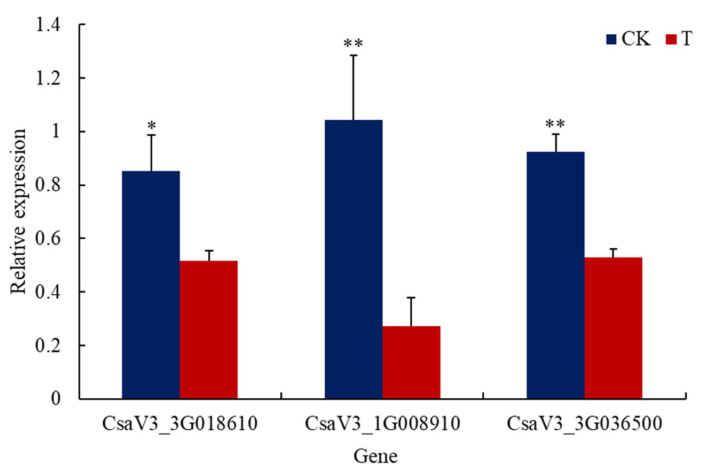
Transcription levels of the representative DEGs related to nitrogen metabolism verified by qRT-PCR analysis. The abscissa represents several down-regulated genes related to nitrogen metabolism, and the ordinate shows the relative expression levels of DEGs. A single asterisk (*) indicates that there is a significant difference in gene expression at the transcription level between the T group and the CK group (*p* < 0.05). The double asterisks (**) mean that there is an extremely significant difference in gene expression at the transcription level between the T group and the CK group (*p* < 0.01). T: cucumber roots treated with the bacterial cells of GXGL-4A; CK: cucumber roots treated with sterile water (the control).

The transcriptome analysis of *C*. *sativus* root showed that most of the genes related to nitrate transport such as the genes *Csa2G416080* and *Csa1G047430* encoding nitrate transporter (NRT) had no significant difference at the transcription level between groups (*p* > 0.05). However, the transcription levels of NRT-encoding genes *Csa5G161290* and *Csa3G027720* in cucumber roots treated with the bacterial cells of GXGL-4A were significantly enhanced compared to those of the CK group (*p* < 0.05, Figure 6). Additionally, the genes encoding the nitrate reductase (*CsaV3_3G036500*), high-affinity nitrate transporter 3.1-like (*CsaV3_1G008910*), and the ferredoxin-nitrite reductase (*CsaV3_3G018610*) showed a significant down-regulation at the transcription level (*p* < 0.05, Figure 7).

### 3.10. Relative Transcription Levels of the Functional Genes Related to Hormone Synthesis

The results of transcriptome analysis showed that, when treated with GXGL-4A, a gene *CsaV3_5G006200* encoding cytokinin dehydrogenase that is involved in the zeatin biosynthesis and one brassinosteroid biosynthesis gene *CsaV3_3G034190* encoding 3-oxo-5-alpha-steroid 4-dehydrogenase of *C*. *sativus* would be significantly up-regulated at the transcription level (*p* < 0.05). Especially, it was noticed that the transcription of gene *CsaV3_3G034190* was not detected in the CK group (Table S2).

### 3.11. Relative Transcription Levels of the Functional Genes Related to Abiotic Stress Response

**Figure 8:**
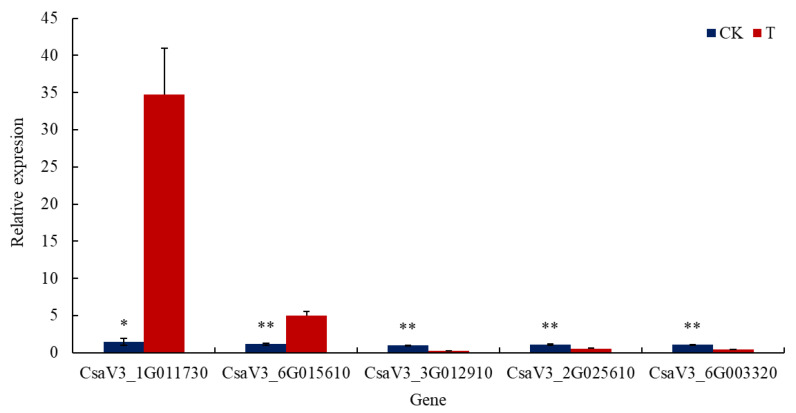
Transcription levels of functional genes related to abiotic stress response in the cucumber roots between the T and CK groups. A single asterisk (*) means that there is a significant difference at the transcription level (*p* < 0.05). Double asterisks (**) represent an extremely significant difference at the transcription level (*p* < 0.01). T: cucumber roots treated with the bacterial cells of GXGL-4A; CK: cucumber roots treated with sterile water (the control).

The relative transcription levels of some genes associated with abiotic stress response in cucumber roots varied significantly after GXGL-4A treatment. The transcription levels of genes *CsaV3_1G011730* and *CsaV3_6G015610*, respectively, encoding a wound-responsive protein and a heat stress transcription factor A-4c-like exhibited significant up-regulation compared to those of the CK group (*p* < 0.05). However, the transcription level of gene *CsaV3_3G012910* encoding a heat stress transcription factor A-6b displayed significant down-regulation (*p* < 0.05, Figure 8). The transcription differences of these genes stated above were further validated by qRT-PCR analysis.

## 4. Discussion

The absorption and utilization of nitrogen are mainly accomplished through nitrate transporters. Gene *Csa1G047430* belongs to NRT2 family, which could be induced by nitrate [33]. In the experiment, the transcription of *Csa1G047430* was significantly increased after treatment with the NFB bacterial strain GXGL-4A. The genes *Csa2G416080*, *Csa5G161290*, and the gene *Csa3G027720* belong to the sole dual-affinity nitrate transport system NRT1.1 in NRT1 family. A low concentration of nitrate in plant roots could stimulate the expression of AtNRT1.1. When the nitrate content was sufficient, the expression of NRT1.1 gene would be down-regulated. In the absence or low concentration of nitrate, the expression of NRT1.1 gene would increase [8,34]. The transcription levels of NRT1.1 genes *Csa3G027720* and *Csa5G161290* were significantly enhanced after the application of GXGL-4A (*p* < 0.05), but the difference in transcription level of NRT1.1 gene *Csa2G416080* was not statistically significant among groups. The underlying reason is unclear. The bacterial cells of GXGL-4A can provide nitrogen nutrition for cucumber plant growth by BNF. The nitrogen source in the Hoagland nutrition solution was ammonium nitrogen resulting from the BNF of GXGL-4A, and there was no nitrate nitrogen in the culture solution; thus, cucumber seedlings grew under the stress conditions of nitrate nitrogen deficiency. *Csa2G416080* was not induced by GXGL-4A treatment, whereas *Csa3G027720* and *Csa5G161290* were up-regulated, suggesting these genes might have diverse roles in nitrogen transportation. Similar results were reported in NRT1 genes *MeNPF5.4* and *MeNPF6.2* in response to nitrogen stress in rice. *MeNPF5.4* was not induced by nitrogen treatment; however, *MeNPF6.2* was up-regulated in high nitrogen treatment [35]. The ammonium produced by NFB is secreted into the soil and, subsequently, can be converted into nitrate that is easily absorbed by plants by nitrifying bacteria in the soil. The NRT1 family related to nitrogen uptake and utilization is important for the improvement of nitrogen use efficiency in crops. NRT1.1 family genes were widely distributed in cucumber plants and had many functions. Studies have shown that *Arabidopsis* NRT1.1 could not only participate in dual-affinity nitrate transport but also was involved in the regulation of root auxin [36,37]. Therefore, it may be considered that the application of NFB can improve the nitrogen utilization efficiency in plants.

The transcription level of WAT1 gene that is involved in the transport of auxin in plant vacuoles was significantly down-regulated, but most genes related to auxin metabolism were significantly increased (*p* < 0.05). Since NFB treatment leads to the enhancement of the transcription levels of multiple genes involved in the biosynthesis or regulation of plant growth hormones, it could be deduced that the promoting effect of GXGL-4A on cucumber plants may be partially attributed to the alteration of endogenous plant hormone profiles.

Zeatin, a cytokinin first isolated from the endosperm of Indian corn, can promote cell division, and biosynthesis and metabolism studies of zeatin have been conducted during the past decades [38,39]. The transcription level of gene *CsaV3_5G006200* encoding cytokinin dehydrogenase that is related to zeatin biosynthesis was significantly increased in cucumber roots after treatment with GXGL-4A, suggesting an improvement in zeatin synthesis.

Brassinosteroids (BRs) are steroid hormones that are essential for plant growth and development. Responses to these hormones are mediated by transcription factors of the bri1-EMS suppressor 1/brassinazole resistant 1 subfamily, and BRs activate these factors by impairing their inhibitory phosphorylation by GSK3/shaggy-like kinases [40]. Brassinosteroid regulates seed size and shape in *Arabidopsis* [41]. Recently, scientists revealed that Brassinosteroid-mediated reactive oxygen species were essential for tapetum degradation and pollen fertility in tomato [42].

Several ethylene-responsive transcription factors such as ERF019 (*CsaV3_2G033120*) exhibited a significantly up-regulated response. The ERF019 in *Arabidopsis thaliana*, AtERF019, negatively regulates plant resistance to *Phytophthora parasitica* by suppressing PAMP-triggered immunity, suggesting the importance of ERF019 in mediating plant susceptibility to phytopathogens through suppression of pathogen-associated molecular-pattern-triggered immunity (PTI) [43]. On the other hand, the transcription levels of genes, *CsaV3_1G044030* and *CsaV3_6G042970*, respectively, encoding a 23 kDa jasmonate-induced protein-like (JIP23-like) and a salicylic acid-binding protein 2 (SABP2) significantly decreased (Table S2). In excised leaf tissues of barley, as one major jasmonate effect, the induction of novel abundant proteins designated as jasmonate-induced proteins (JIPs) was observed, and JIP23 has been implicated in translational control [44,45]. High-affinity SABP2 was required for plant innate immunity and had salicylic-acid-stimulated lipase activity [46].

To adapt to microbial habitats and resist phytopathogens, plants evolved two defense systems, pattern-triggered immunity (PTI) and effector-triggered immunity (ETI) [47]. Plants triggered PTI by recognizing pathogen-associated molecular patterns (PAMPs) and pattern recognition receptors (PRRs). The plant-encoded R protein and the corresponding pathogenic effector protein Avr occurring directly or indirectly trigger a defense response; this is the effector-triggered immunity (ETI). Many studies showed that ethylene, salicylic acid, and jasmonic acid were related to plant disease resistance. Salicylic acid (SA) could induce the expression of a variety of disease-related protein genes to resist disease. SA participates in the process of PTI and ETI and could also cause hypersensitivity reactions in plants. Jasmonic acid (JA) was essential for plants to cope with biological stress [48]. Ethylene was the only gaseous hormone in plants and played a vital role in the growth, development, and resistance of plants [49]. In most cases, the ethylene and JA signaling pathways together activated the expression of disease resistance genes [50,51,52]. Ethylene could also regulate the antagonism between SA and JA signaling pathways by enhancing the disease resistance response mediated by salicylic acid/NPR1 to increase plant resistance [53]. In this study, five ethylene-related transcription factors showed a significant up-regulation at the transcription level in the cucumber root when treated with the NFB strain GXGL-4A, and the transcriptions of JA- and SA-related genes significantly decreased (Table S2).

Mitogen-activated protein kinase (MAPK) cascades are highly conserved signaling modules that co-ordinate diverse biological processes such as plant innate immunity and development [54]. In this work, six DEGs were enriched in mitogen-activated protein kinases (MAPK) signaling pathway, but the enrichment did not reach a significant level. To our knowledge, to date, there is no direct evidence that the MAPK cascades play an essential role in nitrogen metabolism. Nevertheless, it is reported that MAPK cascades could be regulating nitrogen assimilation, since the phosphorylation of nitrate reductase (NR) by MAPK6 promotes NO production in *Arabidopsis thaliana* [55]. A recent study revealed that exogenous NO can mitigate oxidative damage and improve nitrogen metabolism in tomato seedlings under low nitrogen stress, and the MAPK signaling pathway was involved in this process [56]. Some researchers found that the genetic expressions of MAPKK kinases (MAPKKKs) RAF14 and RAF79 showed a very strong repression by ammonium in the green microalga *Chlamydomonasreinhardtii*, which suggests that they may have a key role in the regulation of nitrogen assimilation, encouraging further analyzing in detail the role of MAPK cascades in the regulation of nitrogen metabolism [57].

Phenylpropanoids contribute to all aspects of plant responses towards biotic and abiotic stimuli. They are not only indicators of plant stress responses upon variation in light or mineral treatment but are also key mediators of the plant’s resistance towards pests [58]. The general phenylpropanoid metabolism generates an enormous array of secondary metabolites based on the few intermediates of the shikimate pathway as the core unit [59]. Here, the results of KEGG pathway enrichment for analysis of *C*. *sativus* DEGs showed that only the phenylpropanoid biosynthesis pathway was significantly enriched among the KEGG pathways of all DEGs.

Plant–diazotroph interactions have been explored for over a century as a nitrogen source for crops to improve agricultural productivity and sustainability [60]. Diazotrophs that inhabit the plant rhizosphere substantially contribute to nitrogen input in a terrestrial ecosystem. Different plant species provide heterogeneous habitats for rhizosphere diazotrophs by releasing root exudates containing potential resources for microbial utilization. Recently, scientists found that conifer and broadleaf trees showed a strong co-evolution with rhizosphere diazotrophic microbiome, and the identity of tree species impressed the assembly of diazotrophic communities in its rhizosphere [61]. We believe that exploring the effect of NFB GXGL-4A inoculation on cucumber root exudates using metabolomics technology will be an important research topic in the future.

## 5. Conclusions

The NFB strains from non-leguminous plants have been widely used as an effective component of plant growth-promoting agent in agricultural production, but the effects of NFB on the transcriptome of crop root are rarely reported. Here, the DEGs profile in *C*. *sativus* roots treated with the bacterial cells of *K*. *radicincitans* GXGL-4A was detected in the root of *C*. *sativus* cultivar “Xintaimici” through transcriptome analysis. Further verification of some DEGs was conducted using qRT-PCR. The results showed that the transcription levels of NRT1.1-related genes were significantly increased in cucumber roots after treatment with GXGL-4A cells. The transcription levels of a few DEGs involved in the biosynthesis of zeatin and brassinosteroid, nitrate reduction, and plant resistance to abiotic stresses such as heat and wound significantly increased. The results of KEGG pathway enrichment analysis of *C*. *sativus* DEGs revealed that the phenylpropanoid biosynthesis pathway was significantly enriched, suggesting multiple aspects of cucumber plant responses towards the biotic stimulation from GXGL-4A. It was speculated that the application of NFB bacterial cells of GXGL-4A can not only provide an ammonium nitrogen resource for the growth of cucumber seedlings but also plays an important role in regulating the biosynthesis of some endogenous plant hormones. The research findings contribute to a better understanding of the interaction between NFB and plants and should be of benefit for the exploration of the genome-wide transcription patterns of plant roots in response to NFB inoculation and will facilitate penetrating the promoting mechanisms of NFB application on plant growth. Furthermore, these results can provide theoretical guidance for the efficient utilization of NFB strain GXGL-4A as a bioagent in sustainable agricultural production.

## Figures and Tables

**Table 1:** Primers’ sequences used for qRT-PCR in this study.

Primer Name	Primer Sequence (5′ to 3′)	Product Size (bp)
ACT-F	TTCTGGTGATGGTGTGAGTC	151
ACT-R	GGCAGTGGTGGTGAACATG	
*Csa1G047430*-F	GCATCGTGGAAAAGCAGTACA	233
*Csa1G047430*-R	GAAGCAAGCGGAGGCAATA	
*Csa3G027720*-F	GCCACATCGGAAAATCGTT	158
*Csa3G027720*-R	AATTCGTTGTAATGGGGTAAGG	
*Csa5G161290*-F	CTCCGACCGCCAAAATG	240
*Csa5G161290*-R	TCCAAGTGACCCAATGCTAAT	
*Csa2G416080*-F	AGTATCGGGTCGTTGTTTGC	162
*Csa2G416080*-R	AGGGCTTCCTCTTGGCTTT	
*CsaV3_5G006200*-F	TGACCTCTTTTACAGTGTTCTTGG	178
*CsaV3_5G006200*-R	GTAGTCGAATGTATTTTGTGCAGAT	
*CsaV3_1G044030*-F	ACTGAGATTCGTGAACAGGGAC	113
*CsaV3_1G044030*-R	TTTGATAGACATCCGTTCCACTT	
*CsaV3_1G011730*-F	GAATCATCCTCAAATCAGACGG	107
*CsaV3_1G011730*-R	CCCCAGCAGCTCAAATACATA	
*CsaV3_6G015610*-F	GGGAAAGTGAATGAGTTCTTAGTTG	151
*CsaV3_6G015610*-R	GACCTACATTACTCAGCAAATCCTC	
*CsaV3_3G012910*-F	TGATGGCAGAGGTAGTGAAGC	169
*CsaV3_3G012910*-R	TTTTGGTGGATTAATTGTTGGA	
*CsaV3_6G003320*-F	GGGGCATCGTCGTCTTCA	220
*CsaV3_6G003320*-R	TCATCAATTCTCTTATTTGCTTCG	
*CsaV3_2G025610*-F	CTTTCTACGACTCACAACTCTCAAC	127
*CsaV3_2G025610*-R	GATGCTGCTGCTGCTGCT	
*CsaV3_3G018610*-F	TGTCTCCTCCTGTAGCCTCG	160
*CsaV3_3G018610*-R	CCTTCTCTAAACTTCTCCTTCACC	
*CsaV3_1G008910*-F	GAAGAAGTGGCGTACGGTCA	114
*CsaV3_1G008910*-R	ACTGAAGCAAGCGGAGGC	
*CsaV3_3G036500*-F	TCAAATAGTAATCCGTCATCATCAC	190
*CsaV3_3G036500*-R	GCGTTGGTGAATCTCAGTCC	

**Table 2:** COG functional classification of *C*. *sativus* DEGs.

Category	Type	Functional Description	Gene Number
Information storage and processing	A	RNA processing and modification	3
Metabolism	C	Energy production and conversion	5
Cellular processes and signaling	D	Cell cycle control, cell division, chromosome partitioning	1
Metabolism	E	Amino acid transport and metabolism	5
Metabolism	F	Nucleotide transport and metabolism	4
Metabolism	G	Carbohydrate transport and metabolism	14
Metabolism	H	Coenzyme transport and metabolism	5
Metabolism	I	Lipid transport and metabolism	9
Information storage and processing	J	Translation, ribosomal structure, and biogenesis	6
Information storage and processing	K	Transcription	23
Information storage and processing	L	Replication, recombination, and repair	4
Cellular processes and signaling	M	Cell wall/membrane/envelope biogenesis	4
Cellular processes and signaling	O	Post-translational modification, protein turnover, chaperones	20
Metabolism	P	Inorganic ion transport and metabolism	6
Metabolism	Q	Secondary metabolites biosynthesis, transport, and catabolism	6
Poorly characterized	S	Function unknown	195
Cellular processes and signaling	T	Signal transduction mechanisms	7
Cellular processes and signaling	U	Intracellular trafficking, secretion, and vesicular transport	8
Cellular processes and signaling	V	Defense mechanisms	1

## Data Availability

The original contributions presented in the study are included in the article/Supplementary Materials; further inquiries can be directed to the corresponding author. The RNA-seq transcriptome data of the *C*. *sativus* roots are available at the National Center for Biotechnology Information (NCBI) Sequence Read Archive BioProject Database (PRJNA766832).

## Supplementary Materials

The following supporting information can be downloaded at: https://www.mdpi.com/article/10.3390/microorganisms13030506/s1. Figure S1: Functional annotation statistics of *C*. *sativus* unigenes. The abscissa represents the database name, and the ordinate indicates the number or percentage of sequences annotated to the database. Figure S2: Length distribution of assembled *C*. *sativus* transcripts. The abscissa shows the transcript length range and the ordinate exhibits the number of transcripts within the length range of the transcript. Figure S3: Expression distribution of the detected unigenes and transcripts. The abscissa displays the sample names, and the ordinate indicates the expression levels of unigenes and transcripts detected in this study. The log10 logarithm processed values are taken. Each color in the figure represents a sample, and most of the swelling in the figure indicates the region with the most concentrated gene expression in the sample. The picture shows the expression distribution of the detected unigenes and transcripts in each sample of the treatment group (T group) and the control group (CK group). Table S1: Annotation statistics of the unigenes and transcripts. Table S2: List of the significantly differentially expressed genes (DEGs) in this study.
