# Supplementary material for: Short-Term Fertilization with the Nitrogen-Fixing Bacterium (NFB) Kosakonia radicincitans GXGL-4A Agent Can Modify the Transcriptome Expression Profiling of Cucumber (Cucumis sativus L.) Root
Source: Microorganisms. 2025 Feb 25;13(3):506. doi: 10.3390/microorganisms13030506 (PMC11945905; doi:10.3390/microorganisms13030506)
Supplement: Supplementary file 1 [file microorganisms-13-00506-s001.zip › Table S1.pdf]

Table S1 Annotation statistics of the unigenes and transcripts

Database	Expressed gene number (percent)	Expressed transcript number (percent)	Number of total genes (percent)	Number of total transcript (percent)
GO	16765 (0.7924)	30759 (0.7975)	18656 (0.7349)	33678 (0.7637)
KEGG	8285 (0.3916)	16678 (0.4324)	8975 (0.3535)	17939 (0.4068)
COG	16387 (0.7745)	31482 (0.8163)	17673 (0.6961)	33759 (0.7656)
NR	20283 (0.9586)	37340 (0.9681)	23887 (0.9409)	42186 (0.9567)
Swiss-Prot	15841 (0.7487)	30266 (0.7847)	17205 (0.6777)	32635 (0.7401)
Pfam	16563 (0.7828)	30970 (0.803)	17979 (0.7082)	33384 (0.7571)
Total annotation	20306 (0.9597)	37369 (0.9689)	23922 (0.9423)	42230 (0.9577)
Total	21158 (1.0)	38569 (1.0)	25387 (1)	44097 (1)
