# Supplementary figures and images for: Short-Term Fertilization with the Nitrogen-Fixing Bacterium (NFB) Kosakonia radicincitans GXGL-4A Agent Can Modify the Transcriptome Expression Profiling of Cucumber (Cucumis sativus L.) Root
Source: Microorganisms. 2025 Feb 25;13(3):506. doi: 10.3390/microorganisms13030506 (PMC11945905; doi:10.3390/microorganisms13030506)

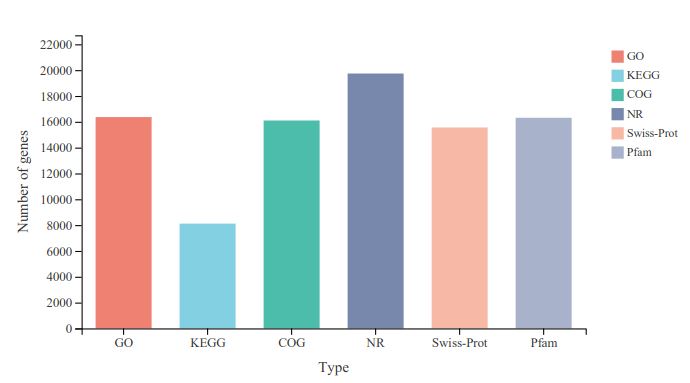

Supplement: Supplementary file 1 [file microorganisms-13-00506-s001.zip › Figure S1.JPG]

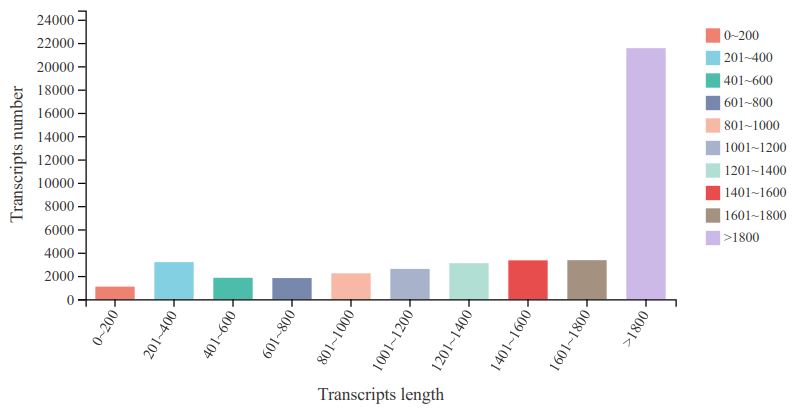

Supplement: Supplementary file 1 [file microorganisms-13-00506-s001.zip › Figure S2.JPG]

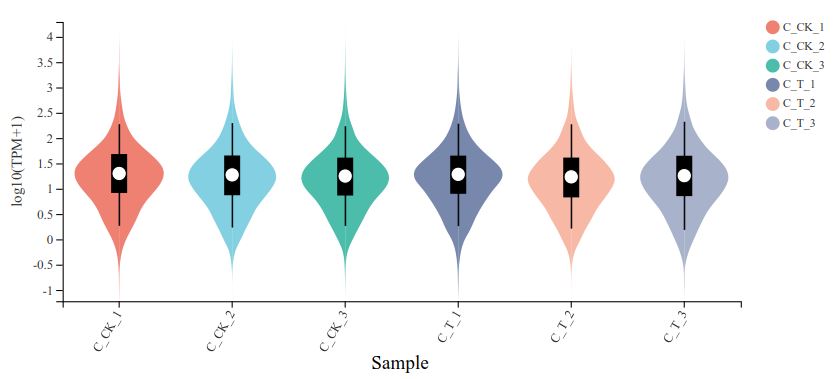

Supplement: Supplementary file 1 [file microorganisms-13-00506-s001.zip › Figure S3.JPG]
